# Mitral regurgitation quantification by cardiac magnetic resonance imaging (MRI) remains reproducible between software solutions

**DOI:** 10.12688/wellcomeopenres.17200.3

**Published:** 2023-05-10

**Authors:** Ciaran Grafton-Clarke, George Thornton, Benjamin Fidock, Gareth Archer, Rod Hose, Rob J. van der Geest, Liang Zhong, Andrew J. Swift, James M. Wild, Estefania De Gárate, Chiara Bucciarelli-Ducci, Sven Plein, Thomas A. Treibel, Marcus Flather, Vassilios S. Vassiliou, Pankaj Garg

**Affiliations:** 1 Medical School, University of East Anglia, Norwich, UK; 2 Institute for Cardiovascular Sciences, University College London Hospitals NHS Trust, London, UK; 3 Department of Infection, University of Sheffield, Sheffield, UK; 4 Department of Radiology, Leiden University Medical Center, Leiden, The Netherlands; 5 National Heart Centre, Duke NUS Graduate Medical School, Singapore, Singapore; 6 British Heart Institute, Britsol, UK; 7 Royal Brompton & Harefield NHS Trust, Guy's and St Thomas' NHS Foundation Trust, London, UK; 8 Leeds Institute of Cardiovascular and Metabolic Medicine, University of Leeds, Leeds, UK

**Keywords:** Magnetic resonance imaging; Mitral valve insufficiency; Reproducibility of results.

## Abstract

**Background: **The reproducibility of mitral regurgitation (MR) quantification by cardiovascular magnetic resonance (CMR) imaging using different software solutions remains unclear. This research aimed to investigate the reproducibility of MR quantification between two software solutions: MASS (version 2019 EXP, LUMC, Netherlands) and CAAS (version 5.2, Pie Medical Imaging).

**Methods:** CMR data of 35 patients with MR (12 primary MR, 13 mitral valve repair/replacement, and ten secondary MR) was used. Four methods of MR volume quantification were studied, including two 4D-flow CMR methods (MR _MVAV_ and MR _Jet_) and two non-4D-flow techniques (MR _Standard_ and MR _LVRV_). We conducted within-software and inter-software correlation and agreement analyses.

**Results:** All methods demonstrated significant correlation between the two software solutions: MR _Standard _(r=0.92, p<0.001), MR _LVRV _(r=0.95, p<0.001), MR _Jet _(r=0.86, p<0.001), and MR _MVAV _(r=0.91, p<0.001). Between CAAS and MASS, MR _Jet_ and MR _MVAV_, compared to each of the four methods, were the only methods not to be associated with significant bias.

**Conclusions:** We conclude that 4D-flow CMR methods demonstrate equivalent reproducibility to non-4D-flow methods but greater levels of agreement between software solutions.

## Introduction

Mitral regurgitation (MR) is one of the most common types of valvular heart disease and is one of the most frequent indications for valve surgery ^1^. Even though echocardiography remains the first-line investigation for MR assessment ^2^, recent evidence suggests that cardiovascular magnetic resonance (CMR) quantitative assessment of MR is more precise and has a better prognostic association ^3^. One of the key strengths of CMR quantification of MR is that it allows many different ways to quantify MR ^4^. These include direct and indirect methods using standard techniques and emerging four-dimensional (4D) flow methods ^5^.

Our recent work demonstrated that 4D-flow methods of MR quantification may offer superior precision for reproducibility compared to standard methods ^5^. In practice, a combination of standard and 4D-flow methods of MR quantification can be used to build confidence in reporting CMR images and clinical decision-making. Our previous work involved the use of a research software solution from Leiden lab (MASS). MASS is not currently a commercial software package for clinical use and is limited to research applications only. Moreover, there is a paucity of evidence evaluating the reproducibility of MR volume quantification between different software solutions across the breadth of methods ^6^. Demonstrating reproducibility between different software solutions is vital as clinical outcome research within CMR imaging is multiplatform and multicentre. It is essential that the data generated from analysis is accurate, precise, and reproducible, regardless of which software platform is used.

The primary objective of this research was to investigate the reproducibility and agreement in MR volume quantification between two software solutions (CAAS, version 5.2, Pie Medical Imaging) using subjects from previously published cohorts spanning the spectrum of MR disease states ^5^. Using CAAS, we also conducted within-software agreement analysis between different methods of MR volume quantification. Third, we present interobserver reproducibility analysis within CAAS across the four methods of MR volume quantification.

## Methods

### Study population

The subjects included within this study have been reported on in other published works ^5^. In brief, the data relates to a UK multicentre prospective study involving 35 subjects with MR diagnosed on echocardiography. Recruited from outpatient cardiology clinics at two centres with dedicated mitral valve services (Sheffield and Leeds) between January 2015 – December 2020, 12 subjects had primary MR, ten subjects had secondary MR, and 13 subjects had mitral valve replacement (MVR). Patients with significant valvular stenosis and cardiac shunts were not considered eligible. 

### Ethics

This study was approved by the National Research Ethics Committee in the UK (17/LO/0283 and 12/YH/0169). Informed written consent was obtained from all subjects before participation.

### CMR protocol

At Sheffield, CMR was performed on a 3.0 Tesla Phillips Healthcare system (Achieva TX) equipped with a 28-channel coil and Philips dStream digital broadband MR architecture technology. In Leeds, CMR was performed on a 1.5 Tesla Philips Healthcare system (Ingenia Phillips, Best, The Netherlands) with a phased array 28-channel cardiac receiver coil).

The CMR protocol included baseline surveys, cines (vertical long-axis, horizontal long-axis, short-axis contiguous left ventricle volume stack, 3-chamber, and aortic root) and 4D-flow acquisition. Cine images were acquired during end-expiratory breath-holds with a balanced steady-state free precession, single-slice breath-hold sequence. Procedures relating to 4D-flow pre-processing were delivered in accordance with established standards of practice ^7^.

### Image analysis

Image analysis was completed within two CMR software solutions: MASS software (version 2019 EXP, LUMC, Netherlands) and CAAS MR Solutions (version 5.2). The image analysis and MR quantification methods for the MASS platform are published elsewhere ^5^. In CAAS, both aliasing correction and phase offset correction were applied.

**Figure 1.**
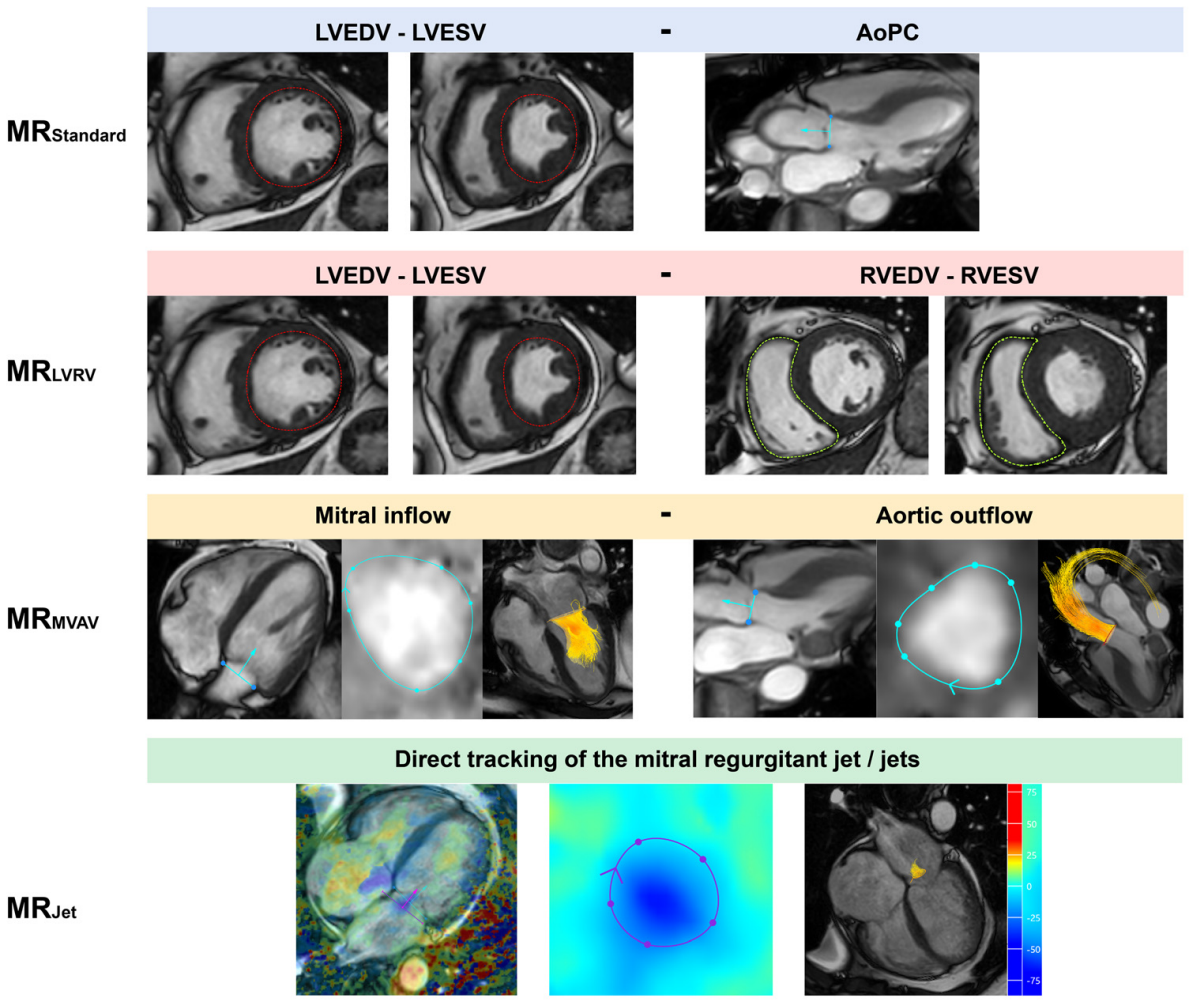
Visual description of the four cardiovascular magnetic resonance imaging mitral regurgitation volume quantification methods investigated within this study. AoPC - aortic phase contrast; LVEDV - left ventricular end diastolic volume; LVESV - left ventricular end systolic volume; RVEDV - right ventricular end diastolic volume; RVESV - right ventricular end systolic volume.

In total, four quantification methods for MR were computed within the CAAS platform, aligning with the methods used within MASS ( Figure 1). One assessor with two years of CMR experience completed the analysis of all 35 subjects within CAAS, blinded to the data generated from MASS.

1. **MR _Standard_** (LVSV - AoPC)
  Left ventricular stroke volume (LVSV) was determined through endocardial segmentation of the short-axis cine stack. Aortic stroke volume was obtained using a static reformatted aortic phase-contrast (AoPC) plane through the sino-tubular junction.

2. **MR _LVRV_** (LVSV - RVSV)
  Right ventricular stroke volume (RVSV) was determined by segmentation of the RV in the short-axis cine stack. This method was not used in the ten patients with secondary MR, given the regular presence of concurrent MR and tricuspid regurgitation.

3. **MR _MVAV_** (4D-flow mitral forward flow - 4D-flow aortic forward flow)
  Using retrospective mitral valve and aortic valve tracking within the four-chamber cine and three-chamber cine, respectively, a phase-contrast, valvular formatted plane was generated. Using the formatted valvular plane, we segmented the forward flow whilst taking into account the through-plane motion of the valve plane.

4. **MR _Jet_** (4D-flow direct jet assessment)
  Jets of MR were directly quantified from the 4D-flow dataset. The jet(s) were first identified in multiple long-axis chamber views. Where available, the four-chamber view was used to draw a reformatted plane perpendicular to the regurgitant jet within the left atrium for each phase it was present. If multiple, jet volumes were summated to provide a total MR volume.

In subjects with previous mitral valve replacement, the prosthetic valve distorts the mitral annulus on four-chamber cines and causes pixelation artefacts in 4D flow imaging around the region of interest. To enable quantification in these subjects, a reformatted plane was placed at the approximate mitral valve location using the tricuspid valve as a reference. For forward flow, the pixel artefact-free slice nearest the mitral valve within the ventricle was used, while for mitral regurgitation, the closest slice within the left atrium without pixel artefacts was utilised.

### Interobserver reproducibility

Interobserver tests were performed by two investigators (CGC, PG) blinded to the results of each other. A random mix of ten subjects was studied, where each investigator estimated MR volume using the four methods previously described. Each observer had at least two years of CMR experience.

### Statistical analysis

All statistical analysis were completed using SPSS version 25, though Microsoft Excel could also be used. All continuous parameters are reported as mean ± standard deviation (SD). Statistical parameters to assess inter-software and within-software MR quantification method correlation were calculated using Pearson correlation coefficient. Agreement between methods of MR quantification within-software (CAAS) and between software’s (CAAS versus MASS) was calculated using Bland-Altman statistics where the mean difference between two methods was reported as the relative risk of bias (measured in ml). For all analyses, p < 0.05 was deemed to be statistically significant. Defined *a priori*, bias between methods of greater than 5 ml was felt to be clinically significant, as determined through consensus amongst study investigators.

## Results

Demographic and clinical data for the 35 subjects are presented in Table 1. Quantification of MR was possible in all subjects, including those with metallic mitral valves. As quantified using CAAS, the average MR volume (across all four methods) for subjects with primary MR was 30.5 ml, 16.4 ml for subjects with secondary MR, and 3.2 ml in those with a replaced/repaired mitral valve.

**Table 1.** Study participant demographics and clinical data.

	Primary MR	Secondary MR	MVR	p-value
**Number of subjects**	12	10	13	-
**Age (years)**	67 ± 11	68 ± 11	62 ± 11	0.97
**Male, n (%)**	6 (50.0)	6 (60.0)	13 (100.0)	0.03
**Height (cm)**	167 ± 8	167 ± 9	177 ± 6	0.04
**Weight (Kg)**	75 ± 11	77 ± 11	93 ± 19	0.01
**Diabetes mellitus (n)**	1	2	1	0.87
**Smoker (n)**	7	7	5	0.98
**Atrial fibrillation (n)**	3	2	0	0.26
**Ischaemic heart disease (n)**	0	0	9	-
**NYHA class**	2.4 ± 0.9	1.3 ± 0.6	1.7 ± 0.7	0.01

MR-magnetic resonance; MRV-mitral valve replacement; NYHA-New York Heart Association.

### Inter-software correlation and agreement

Quantification of MR in CAAS correlated strongly with the values from MASS for all four methods of assessment ( Table 2). MR _LVRV_ was the most strongly correlated method between software solutions (R 0.95, p < 0.001), followed by MR _Standard_ (r = 0.92, p < 0.001) and MR _MVAV_ (r = 0.91, p < 0.001). MR _Jet_ (r = 0.86, p < 0.001) was the least strongly correlated method.

**Table 2.** Correlation and agreement analysis between CAAS and MASS mitral regurgitation quantification methods. Correlation analysis using the Pearson correlation coefficient (denoted *Correlation*) and agreement analysis using Bland-Altman statistics ( *denoted Bias)*. The table provides within-vendor analysis (i.e., correlation and agreement between each method within CAAS software solutions) and inter-vendor analysis (correlation and agreement for each method between CAAS and MASS software solution). For agreement analysis, bias refers to the mean difference between two methods of MR volume quantification (measured in ml) and is deemed statistically significant if the corresponding p-value (denoted P) is < 0.05. For negative bias values, this indicates that the method used in CAAS (uppermost panel) to quantify MR is systematically lower than the method in either CAAS (for within-vendor analysis) or MASS (for inter-vendor analysis). For correlation analysis, a p-value < 0.05 is deemed statistically significant. MR=magnetic resonance.

		CAAS															
		MR _Standard_				MR _LVRV_				MR _MVAV_				MR _Jet_			
		Correlation		Bias		Correlation		Bias		Correlation		Bias		Correlation		Bias	
		R	P	Bias	P	R	P	Bias	P	R	P	Bias	P	R	P	Bias	P
**CAAS**	**MR _Standard_**	-	-	-	-	0.91	<0.001	6.2	0.009	0.85	<0.001	-1.1	0.549	0.73	<0.001	-2.7	0.144
	**MR _LVRV_**	0.91	<0.001	-6.2	0.009	-	-	-	-	0.83	<0.001	-5.2	0.02	0.76	<0.001	-7.2	0.007
	**MR _MVAV_**	0.85	<0.001	1.1	0.549	0.83	<0.001	5.2	0.02	-	-	-	-	0.83	<0.001	-1.7	0.385
	**MR _Jet_**	0.73	<0.001	2.7	0.144	0.76	<0.001	7.2	0.007	0.83	<0.001	1.7	0.385	-	-	-	-
**MASS**	**MR _Standard_**	0.92	<0.001	2.7	0.045	0.89	<0.001	9.1	<0.001	0.86	<0.001	1.7	0.353	0.76	<0.001	0.0	0.988
	**MR _LVRV_**	0.88	<0.001	-4.7	0.064	0.95	<0.001	1.4	0.338	0.85	<0.001	-5.0	0.071	0.78	<0.001	5.8	0.073
	**MR _MVAV_**	0.90	<0.001	3.4	0.006	0.93	<0.001	9.2	<0.001	0.91	<0.001	2.3	0.137	0.75	<0.001	0.6	0.705
	**MR _Jet_**	0.78	<0.001	0.3	0.892	0.81	<0.001	4.3	0.158	0.86	<0.001	-2.1	0.362	0.86	<0.001	2.5	0.169

**Figure 2.**
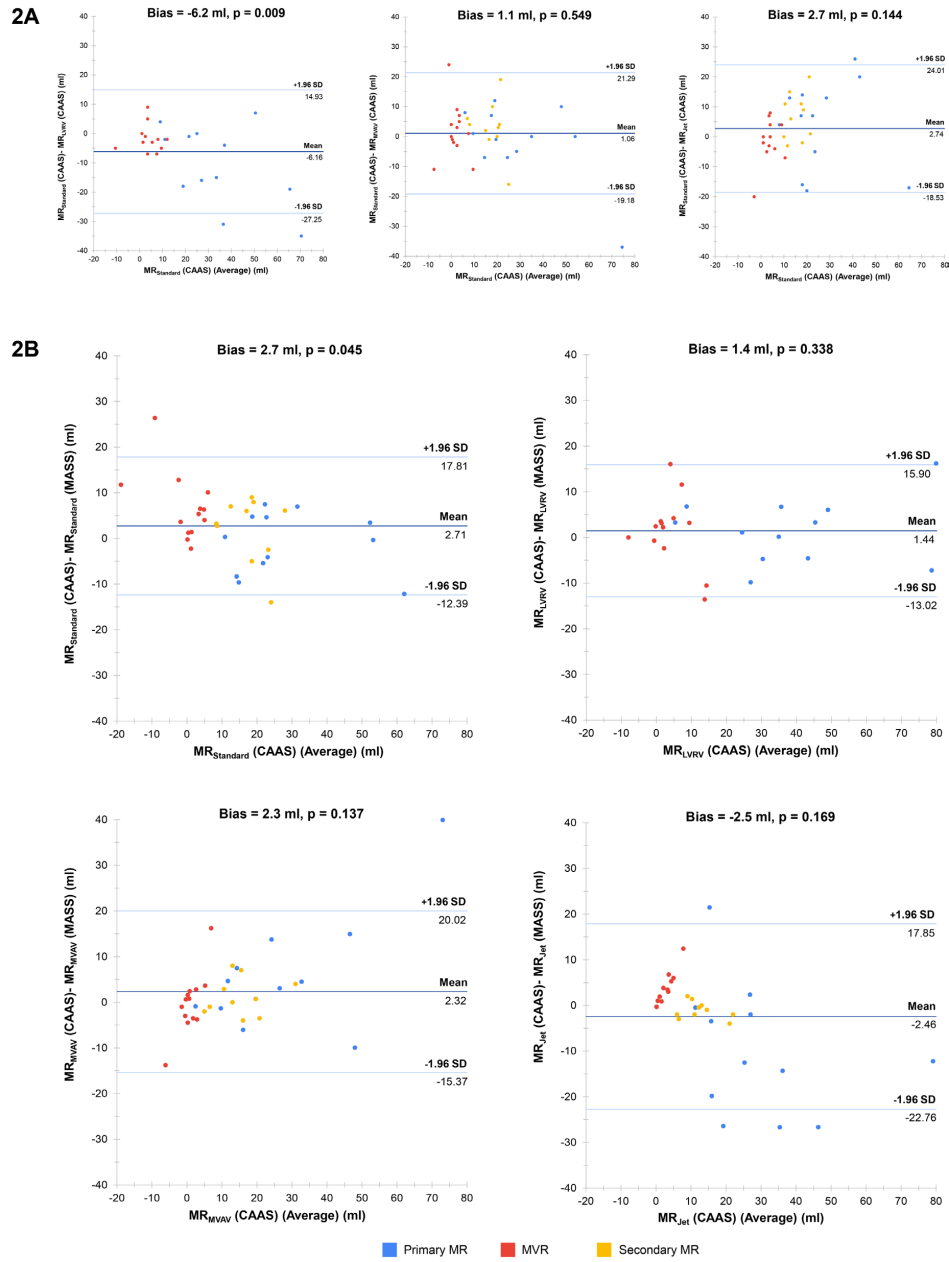
( **A**) Bland-Altman plots for mitral regurgitation within CAAS. Each plot represents a comparison between two methods within CAAS. Bias refers to the mean difference between the methods of mitral regurgitation volume quantification (measured in ml) and is deemed statistically significant (i.e., high risk of systematic bias) if the corresponding p-value is < 0.05. ( **B**) Bland-Altman plots for MR quantification between CAAS and MASS. Each plot represents a comparison between like-for-like methods of mitral regurgitation volume quantification between the two software solutions. MR-magnetic resonance.

Despite being the most strongly correlated method between software solutions, MR _Standard_ was the only method to result in significant bias in agreement between CAAS and MASS MR quantification (bias 2.7 ml, p = 0.045) ( Figure 2). The degree of bias for the other methods was 2.3 ml for MR _MVAV_ (p = 0.137), 1.4 ml for MR _LVRV_ (p = 0.338) and -2.5 ml for MR _Jet_ (p = 0.169). Of note, when we performed subgroup analysis of agreement for the MR _Standard_ stratified by MR type, it was identified that MR _Standard_, when used for MR quantification in subjects with MVR, demonstrated poor levels of agreement (bias 6.7 ml, p = 0.007). This contrasts with the agreement in subjects with primary and secondary MR, where MR _Standard_ was associated with low bias (-1.0 ml, p = 0.604 and 2.1 ml, p = 0.392, respectively). With specific reference to the 4D-flow methods of MR quantification, MR _MVAV_ and MR _Jet_, there was excellent correlation and low bias between these methods in CAAS and all four methods within MASS.

### Within-software correlation and agreement

Using CAAS, we compared each method to each other to determine correlation and agreement/bias. All methods were strongly correlated to each other, with r coefficients ranging from 0.73 to 0.91. Of all method comparisons, MR _Standard_ and MR _LVRV_ were the most positively correlated (r = 0.91 p < 0.001). The least strongly correlated methods were MR _Standard_ and MR _Jet_ (r = 0.73, p < 0.001) and also MR _LVRV_ and MR _Jet_ (r = 0.76, p < 0.001).

Despite being the most strongly correlated, MR _Standard_ and MR _LVRV,_ when compared to each other, were associated with significant levels of agreement bias (6.2 ml, p = 0.009). Further to this, MR _LVRV_ had significant bias when compared to both MR _MVAV_ (5.2 ml, p = 0.020) and MR _Jet_ (7.2, p = 0.007). Aside from comparisons with MR _LVRV_, the 4D-flow methods of MR quantification were associated with low levels of bias when compared to each other and to MR _Standard_.

### Interobserver reproducibility

Reproducibility in analysis between two independent assessors with CAAS demonstrated excellent agreement across all four methods of MR volume quantification ( Table 3). The 4D-flow methods of quantification were the most strongly correlated between observers (MR _Jet_ r = 0.99, p < 0.001; MR _MVAV_ 0.98, p < 0.001). MR _Standard_ and MR _LVRV_ were also strongly correlated (0.96 and 0.94, respectively, p < 0.001). Only MR volume quantification using the MR _Jet_ method between two observers demonstrated significant bias ^8^. MR _Standard_, MR _LVRV_ and MR _MVAV_ methods of quantification were not significantly biased between two observers.

**Table 3.** Interobserver reproducibility analysis.

	Pearson Correlation		Bland Altman	
	r	p-value	Bias (ml)	P-value
**MR _Standard_**	0.964	<0.001	0.2	0.938
**MR _LVRV_**	0.939	<0.001	0.0	1.00
**MR _MVAV_**	0.980	<0.001	0.5	0.789
**MR _Jet_**	0.988	<0.001	-6.6	0.125

Correlation analysis using the Pearson correlation coefficient (denoted *Correlation*) and agreement analysis using Bland-Altman statistics ( *denoted Bias)* between two observers within the CAAS software solution. MR-magnetic resonance.

## Discussion

We have demonstrated that quantification of mitral regurgitation is consistent between two different software solutions. We have also demonstrated that within the CAAS platform, there are high levels of agreement between all methods of quantification. Between software solutions, MR _Standard_ was the only method to result in significant bias and was identified to be due to subjects with mitral valve replacement. We speculate this may be due to the challenges in segmenting the short-axis basal slices in subjects with a MVR. Of note, despite the bias associated with the MR _Standard_ method being determined as statistically significant, the quantity of MR volume of 2.7 ml is not clinically significant.

 Between methods in CAAS, the degree of correlation between all methods was excellent. The MR _Standard_ method was not only strongly correlated with the MR volume quantification methods utilising 4D-flow techniques, but there was a low risk of bias between MR _Standard_ and both MR _MVAV_ and MR _Jet_ methods of quantification. We have therefore demonstrated that within CAAS, with reference to the MR _Standard_ method, agreement is best demonstrated with 4D-flow techniques. We have also shown that between the two software platforms, MR volume quantification using the 4D-flow techniques, is both highly reproducible, and is not associated with significant bias, which was not the case for the non-4D-flow techniques.

A previous multicentre study demonstrated that automated valve tracking on CAAS can provide consistent valvular flow quantification ^9^. Our study complements their work and demonstrates interoperability between different CMR methods of MR quantification. This becomes critically important in routine clinical practice for increasing the confidence of reporting MR severity. In addition, in this study, we have demonstrated agreement and consistency in MR quantification between two software solutions. This is important for the clinical translation of all the methods of MR quantification by CMR described in our study.

Our previous work demonstrated that 4D-flow methods of MR quantification, in particular MR _MVAV_, is superior to other methods of MR quantification for reproducibility as it enhances precision ^5^. As research involving 4D-flow CMR techniques continues to gain interest, there is an evolving need for large multicentre studies with clinical outcomes to provide answers to key clinical questions. It is therefore essential for the research and clinical communities to have confidence that regardless of the software platform used for analysis, the data output is comparable between platforms and can confidently be combined without risk of significant bias.

This study has several limitations. First, patients with MVR and secondary MR only had mild to moderate MR. Second due to lower MR volume in MVR and secondary MR cases, the relative bias may appear larger in Bland-Altman analysis. Third, we have only used one commercially available CMR software for comparison. Fourth, in this cohort, the prevalence of atrial fibrillation was lower than typically observed in standard populations with mitral valve disease. This is noteworthy because the presence of atrial fibrillation can impact image quality, as it complicates the synchronisation of image acquisition with the cardiac cycle. This highlights the need to validate methods of MR quantification is larger, real-world populations. Finally, this study did not evaluate intra-observer variability in MR volume quantification which is an important assessment in ensuring the validity and reproducibility of research findings.

We conclude that 4D-flow CMR methods demonstrate equivalent reproducibility to non-4D-flow methods in the assessment of mitral regurgitation and greater levels of agreement between software solutions. 4D-flow methods of assessment enhance precision of MR quantification and is highly reproducible between different software solutions,

## Consent 

Written informed consent for publication of the participants’ data and data resulting from analysis of their cardiac imaging was obtained from the participants.

## Data Availability

Harvard Dataverse: Mitral regurgitation quantification by cardiac MRI between software solutions. https://doi.org/10.7910/DVN/I8S00H ^8^. This project contains the following underlying data: Data Upload.tab (demographic data; functional data and outputted 4D-flow data from both software solutions; inter-observer data between assessor 1 and 2) Supplemental Material.docx (technical information for 4D-flow echo-planar imaging (EPI) and Cine imaging CMR protocol sequence details). Data are available under the terms of the Creative Commons Zero "No rights reserved" data waiver (CC0 1.0 Public domain dedication). Raw CMR images were not uploaded in order to protect the identity of the subjects. Access can be requested by contacting the corresponding author ( Ciarang-c@hotmail.com). Access to the raw CMR images will be granted for the purpose of re-analysis relating to the primary aims of this research.
