# Supplementary material for: Mitral regurgitation quantification by cardiac magnetic resonance imaging (MRI) remains reproducible between software solutions
Source: Wellcome Open Res. 2023 May 10;6:253. Originally published 2021 Oct 5. [Version 3] doi: 10.12688/wellcomeopenres.17200.3 (PMC10220421; doi:10.12688/wellcomeopenres.17200.3)
Supplement: Peer review history

# Peer review: Reviewer response for version 3

**DOI:** 10.21956/wellcomeopenres.21503.r57154

**Published:** 2023-05-26

**Authors:** Nay Aung

**Affiliations:** 1 William Harvey Research Institute, National Institute for Health and Care Research Barts Biomedical Research Centre, Queen Mary University London, London, UK

I have reviewed the revision version. The authors have addressed my comments and I have no further suggestions. The manuscript is suitable for indexing from my perspective.

Is the work clearly and accurately presented and does it cite the current literature?

Yes

If applicable, is the statistical analysis and its interpretation appropriate?

Yes

Are all the source data underlying the results available to ensure full reproducibility?

Yes

Is the study design appropriate and is the work technically sound?

Yes

Are the conclusions drawn adequately supported by the results?

Yes

Are sufficient details of methods and analysis provided to allow replication by others?

Yes

Reviewer Expertise:

CMR, genomics, AI

I confirm that I have read this submission and believe that I have an appropriate level of expertise to confirm that it is of an acceptable scientific standard.

# Peer review: Reviewer response for version 2

**DOI:** 10.21956/wellcomeopenres.19429.r55967

**Published:** 2023-04-18

**Authors:** Nay Aung

**Affiliations:** 1 William Harvey Research Institute, National Institute for Health and Care Research Barts Biomedical Research Centre, Queen Mary University London, London, UK

This paper by Grafton-Clarke and colleagues nicely compared and contrasted the correlation and agreement of 4 MR quantification techniques (2 conventional and 2 4D-flow) in CMR. They demonstrated excellent correlations of MR volume measures across 4 techniques and low biases, especially with 4D-flow-derived techniques. Given the relative sparsity of evidence around MR quantification with CMR, this work provides additional information and confidence in CMR-based techniques.

 The study methodology is sound and the data is very clearly presented. I have the following comments:

1. Please comment on the issues around image quality (especially with regard to the cases with previous MV replacement). I assume there will be some cases with prosthetic MVR in this cohort - did the metallic artefact cause any issue with any of the techniques described here?
2. Also, it should be noted that given the absence of atrial fibrillation which frequently co-exists with chronic MR during these CMR examinations, this reviewer feels that this study represents the 'best-case' and optimal scenario. This is not an issue for this paper but it should be commented in the limitations or the future work section on the need for validation in larger, real-world cases.
3. In the limitation section, the absence of **intra-observer **variability assessment should be mentioned.  

Is the work clearly and accurately presented and does it cite the current literature?

Yes

If applicable, is the statistical analysis and its interpretation appropriate?

Yes

Are all the source data underlying the results available to ensure full reproducibility?

Yes

Is the study design appropriate and is the work technically sound?

Yes

Are the conclusions drawn adequately supported by the results?

Yes

Are sufficient details of methods and analysis provided to allow replication by others?

Yes

Reviewer Expertise:

CMR, genomics, AI

I confirm that I have read this submission and believe that I have an appropriate level of expertise to confirm that it is of an acceptable scientific standard, however I have significant reservations, as outlined above.

# Peer review: Reviewer response for version 2

**DOI:** 10.21956/wellcomeopenres.19429.r48027

**Published:** 2022-01-27

**Authors:** Antonio Luna

**Affiliations:** 1 Department of Imaging, HT Médica, Jaén, Spain

Dear Authors,

 Thanks for submitting the requested revisions to your research. You have fulfilled most of the changes suggested. In my opinion, it is now ready to be indexed.

 Many thanks.

Is the work clearly and accurately presented and does it cite the current literature?

Yes

If applicable, is the statistical analysis and its interpretation appropriate?

Yes

Are all the source data underlying the results available to ensure full reproducibility?

Yes

Is the study design appropriate and is the work technically sound?

Yes

Are the conclusions drawn adequately supported by the results?

Yes

Are sufficient details of methods and analysis provided to allow replication by others?

Yes

Reviewer Expertise:

Radiologists subspecialist in CMR

I confirm that I have read this submission and believe that I have an appropriate level of expertise to confirm that it is of an acceptable scientific standard.

# Peer review: Reviewer response for version 1

**DOI:** 10.21956/wellcomeopenres.19004.r46923

**Published:** 2021-11-22

**Authors:** Antonio Luna

**Affiliations:** 1 Department of Imaging, HT Médica, Jaén, Spain

Dear Authors,

 Thanks for submitting your research to Wellcome Open Research. You have evaluated the reproducibility of mitral regurgitation using four different methods of CMR from cine and 4D flow acquisitions. For this purpose, you have performed quantifications using a research and commercial software, comparing inter-software and within-software reproducibility, and also interobserver reproducibility. 4D flow methods showed equivalent reproducibility to cine-based ones but with greater levels of agreement. This series adds light to the added value of 4D flow for mitral regurgitation assessment and quantification, supporting similar results using different software platforms. Before indexing, there are some obscure points in the material & methods and results section that should be clarified, as detailed below.

**Material & methods:**

- Define the type of coil and elements used in the 1.5T MR.
- Aortic phase contrast sequences are not included as part of the acquisition protocols. I assume that both pulmonary and aortic valve phase contrast sequences were part of it. Please clarify this point.
- Include a table with the parameters of 4D flow acquisitions, aortic phase contrast sequences and short axis cines in both MRI.
- Define the experience of the assessor completing the analysis of all 35 included patients with CAAS software.
- Interobserver reproducibility was performed in a random mix of ten patients. I suppose that excluding cases with secondary MR, as in those you didn't measure **MR _LVRV_**. Please confirm.

**Results:**

- You state that all patients were in sinus rhythm at the time of CMR acquisition, but in Table 1, 5 patients are described as having AF.

**Discussion:**

- Add a final paragraph with the main conclusion of your research.

Is the work clearly and accurately presented and does it cite the current literature?

Yes

If applicable, is the statistical analysis and its interpretation appropriate?

Yes

Are all the source data underlying the results available to ensure full reproducibility?

Yes

Is the study design appropriate and is the work technically sound?

Yes

Are the conclusions drawn adequately supported by the results?

Yes

Are sufficient details of methods and analysis provided to allow replication by others?

Yes

Reviewer Expertise:

Radiologists subspecialist in CMR

I confirm that I have read this submission and believe that I have an appropriate level of expertise to confirm that it is of an acceptable scientific standard, however I have significant reservations, as outlined above.

# Response

**Published:** 2021-12-29

**Authors:** Ciaran Grafton-Clarke

**Affiliations:** Norfolk and Norwich University Hospitals NHS Foundation Trust, UK

Dear Dr Antonio Luna,

 Thank you for your peer review. We have addressed each of your comments below. 

**Comment 1:** Define the type of coil and elements used in the 1.5T MR.

**Response:** We have added this information within the main manuscript.

**Comment 2: **Aortic phase contrast sequences are not included as part of the acquisition protocols. I assume that both pulmonary and aortic valve phase contrast sequences were part of it. Please clarify this point.

**Response:** The aortic phase contrast study was not done. A 4D flow static 2D phase contrast reformatted plane was used instead. 

**Comment 3:** Include a table with the parameters of 4D flow acquisitions, aortic phase contrast sequences and short axis cines in both MRI.

**Response:** Thank you for this comment. This has now been added to the supplementary document.

**Comment 4: **Define the experience of the assessor completing the analysis of all 35 included patients with CAAS software.

**Response:** Thank you for this comment. This has been inserted into the manuscript.

**Comment 5:** Interobserver reproducibility was performed in a random mix of ten patients. I suppose that excluding cases with secondary MR, as in those you didn't measure **MR _LVRV_**. Please confirm.​​​​​​​

**Response**: Thank you for this comment. This is clarified within the manuscript in the *image analysis *subsection.

**Comment 6:** You state that all patients were in sinus rhythm at the time of CMR acquisition, but in Table 1, 5 patients are described as having AF.

**Response:** Thank you for this comment and we apologise for this inconsistency. This has possibly resulted from the fact that these five patients have had a history of atrial fibrillation, but during the CMR investigation were in sinus rhythm. To avoid confusion, we have removed this comment from the results section.

**Comment 7:** Add a final paragraph with the main conclusion of your research.

**Response: **Thank you for this comment. We have now added this.

 Yours faithfully,

**Dr Ciaran Grafton-Clarke** (corresponding author)

 Dr Pankaj Garg
